# Pressure injury prevalence and characteristics in patients with COVID-19 admitted to acute inpatient rehabilitation unit

**DOI:** 10.3389/fresc.2023.1058982

**Published:** 2023-04-03

**Authors:** Weiying Lu, Ona Bloom, Melissa Rathgeber, Susan Maltser

**Affiliations:** ^1^ Department of Physical Medicine and Rehabilitation, Donald and Barbara Zucker School of Medicine at Hofstra Northwell, Hempstead, NY, United States; ^2^ Institute of Molecular Medicine, The Feinstein Institute for Medical Research, New York, NY, United States; ^3^ Department of Rehabilitation Services, Northwell Health, New York, NY, United States

**Keywords:** COVID-19, pressure injury, rehabilitation coronavirus 2 (SARS-CoV-2), acute rehabilitation humans, quality improvement-outcomes

## Abstract

**Objective:**

To investigate the incidence and severity of pressure injuries among COVID-19 patients who required acute hospitalization and subsequent acute inpatient rehabilitation (AIR).

**Design:**

Data was collected retrospectively from medical charts of COVID-19 patients who were admitted to AIR during April 2020–April 2021.

**Setting:**

Acute Inpatient Rehabilitation at a single hospital in the greater New York metropolitan area.

**Participants:**

Subjects included COVID-19 patients (*N* = 120) who required acute hospitalization and subsequent acute inpatient rehabilitation, of whom 39 (32.5%) had pressure injuries.

**Interventions:**

Not applicable.

**Main outcome measure(s):**

The incidence, location, and severity of pressure injuries in COVID-19 patients, as well as demographic and clinical characteristics of the acute hospitalization.

**Results:**

Among patients who developed pressure injuries, more patients received mechanical ventilation (59% vs. 33%, *P* < 0.05) and tracheostomy (67% vs. 17%, *P* < 0.00001). The lengths of stay were longer in both the intensive care unit (ICU) (34 vs. 15 days, *P* < 0.005), and in acute inpatient rehabilitation (22 vs. 17 days *P* < 0.05).

**Conclusion:**

Pressure injuries were more common in COVID-19 patients who had longer lengths of stay, received mechanical ventilation or tracheostomy, during acute hospitalization. This supports the use of protocols to prioritize pressure offloading in this patient population.

## Introduction

In 2019, the novel coronavirus 2 (SARS-CoV-2) started a global pandemic that remains ongoing. Early in the pandemic, New York City (NYC) and the surrounding metropolitan area experienced an exponential surge of acutely ill COVID-19 patients who required acute hospitalization (1). Patients who experience critical illness have a wide range of disabilities requiring intensive rehabilitation. COVID-19 patients incurred prolonged acute hospitalizations and ICU treatments with subsequent disability necessitating intensive inpatient rehabilitation (2). Between March 2020 and April 2020, out of the 5,700 patients admitted to a NY-based hospital system, 14% were admitted to the intensive care unit (ICU) (3). On discharge, 5.9% of the patients were discharged to a post-acute facility such as acute inpatient rehabilitation (AIR) or skilled nursing facility to address functional decline and to manage ongoing medical needs (3).

Pressure injuries are a potential complication experienced by critically ill patients due to immobility from prolonged hospitalization, direct pressure from positioning during proning and a systemic inflammatory response (4). Pressure injuries can develop through a variety of environmental or physical conditions such as age, smoking, diabetes mellitus, peripheral vascular disease, immobility, and direct pressure (5–7). Because pressure injuries are most commonly caused by immobility or prolonged direct pressure, there is a high incidence of pressure injury development in patients in the ICU (8). Patients who are bedridden have a 5-fold increased risk and patients who required the use of wheelchairs had a 4-fold increased risk of developing pressure injuries (9). Furthermore, medical device related pressure injuries account for approximately 35% of hospital-acquired pressure injuries (HAPI) (10). Mechanical ventilation accounts for 28% of all HAPIs in the critical care patient population (11). The incidence rate of ICU-acquired pressure injuries rate is highly variable and has been reported from 3% to 56% (12, 13).

The goal of this study was to characterize the incidence and severity of pressure injuries among COVID-19 patients who required acute hospitalization and subsequent acute inpatient rehabilitation in the NY metropolitan area during the first surge in 2020.

## Methods

Data was collected retrospectively from medical charts of COVID-19 patients who were admitted during April 2020–April 2021 to Acute Inpatient Rehabilitation (AIR) at a single hospital in the greater New York metropolitan area. Data was collected from electronic health records (Allscripts and Sunrise) in a HIPAA compliant database in RedCap. Inclusion criteria were patients who were admitted sequentially for inpatient rehabilitation after acute hospitalization for COVID-19. Patient records were classified into groups based on the presence or absence of pressure injuries acquired during their acute care hospitalizations. Data collected included: gender, age, race, ethnicity, ICU length of stay, AIR length of stay, rates of mechanical ventilation in the ICU, rates of tracheostomy in the ICU, rates of proning in the ICU, number of pressure injuries, stages of pressure injuries, and location of pressure injuries. Statistical significance was determined using unpaired *T*-tests, with significance *P* < 0.05 using Prism GraphPAD Software. For comparison, historical data was extracted from the Uniform Data System (UDS) for 2019, prior to the COVID-19 pandemic. The numbers of patients that developed pressure injuries obtained from this period of time were from a large sample size of 1,345 patients and thus were able to provide a good estimate of pressure injury incidence pre-COVID-19.

## Results

There were 120 patients who satisfied the inclusion criteria for the study, of whom 39 (32.5%) had pressure injuries. By comparison, UDS data obtained from the same facility in 2019 showed 1.7% patients admitted to AIR had pressure injuries. Clinical and demographic information is provided in Table 1. The age of patients was 56 ± 10.7 in the pressure injury group and 60.6 ± 12.9 in the non-pressure injury group. Age, race and ethnicity were comparable between the two groups. Both groups were predominantly male (Table 1). Length of stay in the ICU was longer for patients with pressure injuries (34 vs. 15 days, *P* < 0.005) (Table 2). Length of stay in acute inpatient rehabilitation was longer for patients with pressure injuries (22 vs. 17 days, *P* < 0.05) (Table 2). The number of patients who received mechanical ventilation was higher in the group that developed pressure injuries (59.0% vs. 33%, *P* < 0.01) (Table 3). Tracheostomy rates were higher for patients with pressure injuries (67% vs. 17%, *P* < 0.00001) (Table 3). Rates of proning were comparable between the groups (Table 3). Of the 70 pressure injuries with recorded location data, most (*N* = 60, 85.7%) had typical pressure injuries in typical locations, while a minority (*N* = 10, 14.3%) had atypical pressure injury locations, such as the face and ear (Table 4) (14).

**Table 1:** Clinical and demographics variables of patients.

	Pressure injury (Yes)	Pressure injury (No)	*P*-value
Age	56.03 (10.73)	60.64 (12.95)	NS
Gender	Male: 32 (82.1%)	Male: 53 (65.4%)	
	Female: 7 (17.9%)	Female: 28 (34.6%)	
Caucasian	14 (35.9%)	29 (35.8%)	
African American	4 (10.3%)	12 (14.8%)	
Asian	7 (17.9%)	15 (18.5%)	
Other	14 (35.9%)	25 (30.9%)	
Hispanic	6 (15.4%)	11 (13.6%)	
Non-hispanic	33 (84.6%)	70 (86.4%)	

For measurements, numbers are written using the following format: mean ± standard deviation and mean (percentage). *P*-value < 0.05 was significant. The *P*-values > 0.05 were designated NS (not significant).

**Table 2:** Lengths of stay in ICU and AIR stay in COVID-19 patients ± pressure injuries.

Length of stay	Did the patient have pressure injuries?	Patients (*N*)	Days (mean ± *σ*)	*P*-value
ICU (days)	No	19	15.42 ± 7.61	*P* = 0.001
	Yes	12	34.42 ± 20.72	
AIR (days)	No	81	16.81 ± 8.30	*P* = 0.03
	Yes	39	21.62 ± 15.71	

*P*-value < 0.05 was significant. The *P*-values > 0.05 were designated NS (not significant).

**Table 3:** Ventilation, tracheostomy, and proning in the ICU of COVID-19 patients ± pressure injuries.

	Patients who received ventilation (*N*, %)	Patients who received tracheostomy (*N*, %)	Patients proned (*N*, %)
Pressure injury-Yes	(23, 59.0%)	(26, 66.7%)	(7, 17.9%)
Pressure injury-No	(27, 33.3%)	(14, 17.3%)	(13, 16.0%)
*P*-value	0.008	*P* < 0.00001	NS

*P*-value < 0.05 was significant. The *P*-values > 0.05 were designated NS (not significant).

**Table 4:** Relative frequencies, locations, and stages of the pressure injuries.

Pressure injury characteristics	Pressure injuries *N* (%)
**Location**	
Face	8 (11.4%)
Ear	2 (2.9%)
Shoulder	1 (1.4%)
Elbow	2 (2.9%)
Back	1 (1.4%)
Hip	5 (7.1%)
Sacral	32 (45.7%)
**Stages**	
I	6 (12%)
II	16 (32%)
III	5 (10%)
IV	9 (18%)
Unstageable	14 (28%)

For measurements, numbers are written using the following format: mean (percentage).

## Discussion

This study examined rates of pressure injury development in COVID-19 patients who were admitted to AIR from April 2020–April 2021. Patients who were admitted with pressure injuries were more likely to have received mechanical ventilation and tracheostomy and had significantly longer length of stay in the ICU and d acute inpatient rehabilitation. Similarly, from April 1 to May 9, 2020, Kendall et al. noted that the prevalence and severity of pressure injuries in inpatient rehabilitation hospital patients increased by 15%, compared to patients admitted before the pandemic (Jan 1–Feb 12, 2020) (15). Sianes-Gallén et al. observed that among 1987 COVID-19 patients, 136 patients (6.8%) developed dependency-related injuries (pressure injuries, moisture-related injuries, friction-related injuries, and skin tears), with 99 of them (73%) being pressure injuries (16). Rrapi et al. identified prolonged lengths of stay and high rates of endotracheal intubation as risk factors of pressure injury development in COVID-19 patients (17).

There are likely several types of risk factors promoting increased incidence and severity of pressure injuries among COVID-19 patients. Molecular mechanisms of tissue injury by SARS-CoV-2 may exacerbate the quantity and severity of the pressure injuries in COVID-10 patients, which may be due in part to the increased risk of thrombosis, microvascular injury, and hypoxemia experienced by COVID-19 patients (16, 18). In addition, SARS-CoV-2 patients are known to have higher activation of systemic inflammation as demonstrated by Zanella et al. (19). Prior to the current pandemic, risk factors that are most associated with increased pressure injury development in the ICU are age, history of diabetes mellitus, define MBP < 60–70 mmHg, mechanical ventilation, and prolonged length of stay (8, 20–22). Between March 1–April 30, 2020, 3,176 out of 11, 542 (27.5%) COVID-19 admissions to acute care hospitals in NY received mechanical ventilation (23). Prone positioning while the patient is undergoing mechanical ventilation is thought to reduce posterior alveoli atelectasis by reducing intrapulmonary shunting and improving ventilation-perfusion matching throughout the lungs (24). However, proning exerts compressive forces on the mouth and nose, increasing the risk of pressure injury to the face by 3-fold (18, 25–27). In the present study, 14.3% of the pressure injuries that had location data were on the face and ears, which are atypical locations for pressure injury development and may have resulted from proning. However, in this study, proning was not statistically significant with regards to pressure injury development, which may have resulted from the limited data available for rates of proning between the two groups of patients.

Also, during the study period, staffing shortages led to hiring of medical personnel with less advanced training, such as medical students as temporary residents, to supplement the medical workforce (28, 29). Furthermore, staffing shortages crossed multiple sectors, as U.S. nursing homes also reported shortages of nurse staff and clinical staff between 2.5% and 18.4% (30). In addition, due to isolation precautions and clinical staff shortages, rehabilitation and mobilization of patients may have been delayed (31). During the first COVID-19 surge (April 2020–2021), AIR units were forced to adapt quickly in order to provide care for a rapidly increasing number of patients. This included changes to infection control policies, space allocation for therapy and staffing modifications (32). Patients in AIR were admitted for a wide range of physical and cognitive disabilities following COVID-19 infection, such as stroke, debility from respiratory failure, vascular complications such as amputation and peripheral neuropathy (2). In this study, patients admitted to AIR with pressure injury had a statistically significant longer length of stay in both the ICU (34 vs. 15 days, *P* < 0.0005) and in acute rehabilitation (22 vs. 17 days, *P* < 0.05) when compared to the group without pressure injury. This may be due in part to the involved care needed to discharge a patient with a wound to the community, as homecare services were limited during the initial surge of COVID-19 patients. Longer lengths of stay in the ICU and AIR may contribute to delay of acute care hospitals to discharge their patients and increased healthcare costs. For example, in 2019, Padula et al. estimated that US hospital-acquired pressure injuries cost $10,708 per patient, and the U.S. had a national average of 2.5 million reported cases, potential costing $26.8 billion annually (33). The COVID-19 pandemic was an unprecedented challenge to the healthcare system as a whole in terms of resource and staff availability (36). During the initial surge of COVID-19 patients, homecare services were limited which may have contributed in part to longer lengths of stay for patients in this study. Given the significant increase in pressure injuries in COVID-19 patients admitted to AIR (32.5% vs. 1.7% of patients during the study period compared to 2019), it was necessary to purchase extra equipment, as well as to expand the wound care treatment team (physicians and nurses) who were able to provide consultations through the use of telehealth (Table 5).

**Table 5:** Recommendations to meet increased needs for wound care among COVID-19 patients.

Purchasing additional ROHO cushions
Purchasing additional Tilt in space wheelchairs
Obtaining additional Clinitron beds
Reliance on RN staff to perform additional wound care treatments
Expansion of wound care to include telehealth consultations

Given hospital ICU surges observed across the U.S. during each wave of the pandemic, developing strategies to prevent pressure injury among patients admitted to ICUs with COVID-19 is warranted. After noting a steady increase of pressure injuries during the first surge (March to May 2020), Polancich et al. observed that prone placement was a significant risk factor for pressure injury development. Thus, they were able to implement interventional strategies, such as placing silicon adhesive dressings on pressure points on the prone patients (34, 35). One such set of recommendations adapted for the care of COVID-19 patients in the acute care setting is summarized in Tables 6, 7, so that similar pandemic surges in the future will have decreased pressure injury incidence (36–39).

**Table 6:** Strategies to decrease pressure injury in critically ill patients.

Supine	Side lying	Prone
Occiput	Side of head	Forehead
Rim of ear	Shoulder	Cheeks
Dorsal thoracic spine	Perineum	Nose
Elbow	Ischium	Chin
Sacrum	Greater trochanter	Clavicle
Coccyx	Anterior knee	Elbow
Heel	Malleolus	Chest/breasts
		Perineum/penis
		Anterior pelvic bones
		Knees/patella
		Dorsal feet/toes

**Table 7:** Recommendations for off-loading of medical devices to decrease pressure injuries in critically ill patients.

Endotracheal tubes
Tracheostomy sites
NG tubes
Oxygen tubing
CPAP/BIPAP mask
O2 saturation probe
Invasive lines
Foley catheter tubing
Intermittent pneumatic compression device tubing

This study had several limitations. First, this was a retrospective study. Second, there was a small sample size (*N* = 120 cases) that satisfied inclusion and exclusion criteria. Third, the comparison of COVID-19 data was to historical data (prior to the COVID-19 pandemic) and not non-COVID-19 patients treated in the same AIR during the same time period. In the future, other covariates such as preexisting comorbidities and time to healing of pressure injury can also be examined to see the effect COVID-19 may or may not have on pressure injuries. Finally, future studies can also examine whether or not having COVID-19 specifically increases the skin to be prone to pressure injury.

## Data Availability

The original contributions presented in the study are included in the article/**Supplementary Material**, further inquiries can be directed to the corresponding author.
