# Supplementary material for: Pressure injury prevalence and characteristics in patients with COVID-19 admitted to acute inpatient rehabilitation unit
Source: Front Rehabil Sci. 2023 Apr 3;4:1058982. doi: 10.3389/fresc.2023.1058982 (PMC10106692; doi:10.3389/fresc.2023.1058982)
Supplement: Supplementary file 2 [file Datasheet2.pdf]

Data Dictionary Codebook

09/30/2022 1:54pm

	#	Variable / Field Name	Field Label<br>*Field Note*	Field Attributes (Field Type, Validation, Choices, Calculations, etc.)
Instrument: **Subject ID and Demographics** (subject_id_and_demographics)				
	1	[record_id]	Record ID	text
	2	[sid]	Site ID:	dropdown<br>1 1<br>2 2<br>3 3<br>4 4<br>5 5<br>6 6
	3	[name]	Name (last, first):	text, Identifier
	4	[unk_1]	If the above question cannot be answered, please check the box here:	checkbox<br>1 unk_1___1 Unknown
	5	[mrn]	MRN:	text (number), Identifier
	6	[unk_2]	If the above question cannot be answered, please check the box here:	checkbox<br>1 unk_2___1 Unknown
	7	[age]	Age	text (number)
	8	[unk_3]	If the above question cannot be answered, please check the box here:	checkbox<br>1 unk_3___1 Unknown
	9	[gender]	Gender:	dropdown<br>1 Male<br>2 Female<br>3 Other
	10	[unk_4]	If the above question cannot be answered, please check the box here:	checkbox<br>1 unk_4___1 Unknown
	11	[race]	Race:	dropdown<br>1 Caucasian<br>2 African American<br>3 Native American<br>4 Asian<br>5 Declined<br>6 Other<br>7 Unknown
	12	[unk_5]	If the above question cannot be answered, please check the box here:	checkbox<br>1 unk_5___1 Unknown
	13	[ethnicity]	Ethnicity:	radio<br>1 Hispanic<br>2 Non-Hispanic
	14	[unk_6]	If the above question cannot be answered, please check the box here:	checkbox<br>1 unk_6___1 Unknown

	15	[ins_adm]	Type of insurance at admission to acute rehab:	dropdown<br>1 Self pay<br>2 Medicare<br>3 Medicaid<br>4 Private<br>5 Uninsured
	16	[unk_7]	If the above question cannot be answered, please check the box here:	checkbox<br>1 unk_7___1 Unknown
	17	[blood]	Blood type:	radio<br>1 A+<br>2 O+<br>3 B+<br>4 AB+<br>5 A-<br>6 O-<br>7 B-<br>8 AB-
	18	[unk_8]	If the above question cannot be answered, please check the box here:	checkbox<br>1 unk_8___1 Unknown
	19	[ht]	Height at admission to acute rehab (cm):	text (number)
	20	[unk_9]	If the above question cannot be answered, please check the box here:	checkbox<br>1 unk_9___1 Unknown
	21	[ht_meters]	Height in meters:	calc<br>Calculation: [ht]*0.01
	22	[wt]	Weight at admission (kg):	text (number)
	23	[unk_10]	If the above question cannot be answered, please check the box here:	checkbox<br>1 unk_10___1 Unknown
	24	[bmi]	BMI:	calc<br>Calculation: [wt]/([ht_meters]*[ht_meters])
	25	[subject_id_and_demographics_complete]	Section Header: *Form Status*<br>Complete?	dropdown<br>0 Incomplete<br>1 Unverified<br>2 Complete
Instrument: **Retrospective Data** (retrospective_data)				
	26	[dx_date]	Section Header: *Data from Acute Care (prior to Acute Rehab stay): NOTE: All data to be entered here will be based on the Acute Care Hospital stay, including inflammatory markers.*<br>COVID-19 diagnosis date:<br>*Upon admission to acute care hospital, or if they developed COVID-19 in the hospital and were tested right away*	text (date_mdy)
	27	[unk_11]	If the above question cannot be answered, please check the box here:	checkbox<br>1 unk_11___1 Unknown
	28	[los_acute]	Length of stay in acute care:<br>*Indicate the number of days stayed*	text (number)
	29	[unk_12]	If the above question cannot be answered, please check the box here:	checkbox<br>1 unk_12___1 Unknown
	30	[icu]	Was this patient transferred to the ICU?	yesno<br>1 Yes<br>0 No
	31	[unk_13]	If the above question cannot be answered, please check the box here:	checkbox<br>1 unk_13___1 Unknown

32	[los_icu]<br>Show the field ONLY if:<br>[icu] = '1'	Length of stay in the ICU:<br>*Indicate the number of days stayed*	text (number)
33	[unk_14]<br>Show the field ONLY if:<br>[icu] = '1'	If the above question cannot be answered, please check the box here:	checkbox<br>1 unk_14___1 Unknown
34	[steroid]	Were systemic steroids administered?	yesno<br>1 Yes<br>0 No
35	[unk_15]	If the above question cannot be answered, please check the box here:	checkbox<br>1 unk_15___1 Unknown
36	[suppress]	Were immunosuppressants (e.g. monoclonal antibody therapies) administered?	yesno<br>1 Yes<br>0 No
37	[unk_16]	If the above question cannot be answered, please check the box here:	checkbox<br>1 unk_16___1 Unknown
38	[mech_vent]	Did the patient receive mechanical ventilation?	yesno<br>1 Yes<br>0 No
39	[unk_17]	If the above question cannot be answered, please check the box here:	checkbox<br>1 unk_17___1 Unknown
40	[mech_vent_type]<br>Show the field ONLY if:<br>[mech_vent] = '1'	Was the ventilation invasive or non-invasive?	radio<br>1 Invasive<br>2 Non-invasive
41	[unk_18]<br>Show the field ONLY if:<br>[mech_vent] = '1'	If the above question cannot be answered, please check the box here:	checkbox<br>1 unk_18___1 Unknown
42	[trach]<br>Show the field ONLY if:<br>[mech_vent_type] = '1'	Did this patient receive a tracheostomy?	yesno<br>1 Yes<br>0 No
43	[unk_19]<br>Show the field ONLY if:<br>[mech_vent_type] = '1'	If the above question cannot be answered, please check the box here:	checkbox<br>1 unk_19___1 Unknown
44	[prone]	Did the patient experience proning?	yesno<br>1 Yes<br>0 No
45	[unk_20]	If the above question cannot be answered, please check the box here:	checkbox<br>1 unk_20___1 Unknown
46	[ecmo]	Was the patient administered ECMO?	yesno<br>1 Yes<br>0 No
47	[unk_21]	If the above question cannot be answered, please check the box here:	checkbox<br>1 unk_21___1 Unknown
48	[ecmo_start]<br>Show the field ONLY if:<br>[ecmo] = '1'	Initial date of ECMO administration?	text (date_mdy)
49	[unk_22]<br>Show the field ONLY if:<br>[ecmo] = '1'	If the above question cannot be answered, please check the box here:	checkbox<br>1 unk_22___1 Unknown
50	[ecmo_dur]<br>Show the field ONLY if:<br>[ecmo] = '1'	What was the duration (in days) of ECMO treatment?	text (number)

51	[unk_23]<br>Show the field ONLY if:<br>[ecmo] = '1'	If the above question cannot be answered, please check the box here:	checkbox<br>1 | unk_23___1 | Unknown
52	[plasma]	Did this patient receive convalescent plasma?	yesno<br>1 | Yes<br>0 | No
53	[unk_24]	If the above question cannot be answered, please check the box here:	checkbox<br>1 | unk_24___1 | Unknown
54	[ferritin]	Peak ferritin level (ng/mL) during their stay:	text (number)
55	[unk_25]	If the above question cannot be answered, please check the box here:	checkbox<br>1 | unk_25___1 | Unknown
56	[crp]	Peak CRP level (mg/dL) during their stay:	text (number)
57	[unk_26]	If the above question cannot be answered, please check the box here:	checkbox<br>1 | unk_26___1 | Unknown
58	[ddimer]	Peak D-dimer level (ng/mL) during their stay:	text (number)
59	[unk_27]	If the above question cannot be answered, please check the box here:	checkbox<br>1 | unk_27___1 | Unknown
60	[il_6]	Peak IL-6 level (pg/mL) during their stay:	text (number)
61	[unk_28]	If the above question cannot be answered, please check the box here:	checkbox<br>1 | unk_28___1 | Unknown
62	[a1c]	Peak HBA1C level (mmol/mol) during their stay:	text (number)
63	[unk_29]	If the above question cannot be answered, please check the box here:	checkbox<br>1 | unk_29___1 | Unknown
64	[adm_date]	Section Header: *Data from Acute Rehab (following initial Acute Care stay):*<br>Admission date to acute rehab:	text (date_mdy)
65	[unk_30]	If the above question cannot be answered, please check the box here:	checkbox<br>1 | unk_30___1 | Unknown
66	[med_cat]	Section Header: *Medications at Admission to Acute Rehab*<br>Please indicate the categories of medication this patient received from the time of admission to acute rehab (examples listed in parenthesis):	checkbox<br>1 | med_cat___1 | Anticoagulants or anti-platelet formation (eliquis, lovenox, xarelto, asa)<br>2 | med_cat___2 | Antiepileptics (keppra, depakote, lamictal)<br>3 | med_cat___3 | Muscle Relaxers<br>4 | med_cat___4 | Neuropathic pain relievers (gabapentin, lyrica, cymbalta)<br>5 | med_cat___5 | Opiates<br>6 | med_cat___6 | Antidepressants (SSRI, trazodone)<br>7 | med_cat___7 | Neuro stimulants (amantadine, ritalin, provigil)<br>9 | med_cat___9 | GI ppx (ppi, H 2)<br>10 | med_cat___10 | Statin<br>11 | med_cat___11 | Antibiotic<br>12 | med_cat___12 | None
67	[unk_31]	If the above question cannot be answered, please check the box here:	checkbox<br>1 | unk_31___1 | Unknown

	68	[impair]	Please indicate the appropriate Impairment code and answer appropriate followup questions according to which codes apply:	checkbox<br>1 impair___1 Stroke<br>2 impair___2 Brain Dysfunction<br>3 impair___3 Neurologic Condition<br>4 impair___4 Non-traumatic Spinal Cord Dysfunction<br>5 impair___5 Traumatic Spinal Cord Dysfunction<br>6 impair___6 Amputation<br>7 impair___7 Arthritis<br>8 impair___8 Pain Syndrome<br>9 impair___9 Orthopedic Disorder<br>10 impair___10 Cardiac<br>11 impair___11 Pulmonary Disorder<br>12 impair___12 Burns<br>13 impair___13 Congenital Deformities<br>14 impair___14 Other Disabling Impairment<br>15 impair___15 Major Multiple Trauma<br>16 impair___16 Developmental Disability<br>17 impair___17 Debility (Non-cardiac/Non-Pulmonary)<br>18 impair___18 Medically Complex<br>19 impair___19 No Impairment
	69	[unk_32]	If the above question cannot be answered, please check the box here:	checkbox<br>1 unk_32___1 Unknown
	70	[impair_stroke]<br>Show the field ONLY if:<br>[impair(1)] = '1'	Stroke, type:	checkbox<br>1 impair_stroke___1 01.1 Left Body Involvement (Right Brain)<br>2 impair_stroke___2 01.2 Right Body Involvement (Left Brain)<br>3 impair_stroke___3 01.3 Bilateral Involvement<br>4 impair_stroke___4 01.4 No Paresis<br>5 impair_stroke___5 01.9 Other Stroke
	71	[unk_33]<br>Show the field ONLY if:<br>[impair(1)] = '1'	If the above question cannot be answered, please check the box here:	checkbox<br>1 unk_33___1 Unknown
	72	[impair_brain]<br>Show the field ONLY if:<br>[impair(2)] = '1'	Brain Dysfunction, type:	checkbox<br>1 impair_brain___1 02.1 Non-traumatic<br>2 impair_brain___2 02.21 Traumatic, Open Injury<br>3 impair_brain___3 02.22 Traumatic, Closed Injury<br>4 impair_brain___4 02.9 Other Brain
	73	[unk_34]<br>Show the field ONLY if:<br>[impair(2)] = '1'	If the above question cannot be answered, please check the box here:	checkbox<br>1 unk_34___1 Unknown
	74	[impair_neuro]<br>Show the field ONLY if:<br>[impair(3)] = '1'	Neurologic Condition, type:	checkbox<br>1 impair_neuro___1 03.1 Multiple Sclerosis<br>2 impair_neuro___2 03.2 Parkinsonism<br>3 impair_neuro___3 03.3 Polyneuropathy<br>4 impair_neuro___4 03.4 Guillain-Barre Syndrome<br>5 impair_neuro___5 03.5 Cerebral Palsy<br>6 impair_neuro___6 03.8 Neuromuscular Disorders<br>7 impair_neuro___7 03.9 Other Neurologic

	75	[unk_35]<br>Show the field ONLY if:<br>[impair(3)] = '1'	If the above question cannot be answered, please check the box here:	checkbox<br>1 unk_35___1 Unknown
	76	[impair_nt_scd]<br>Show the field ONLY if:<br>[impair(4)] = '1'	Non-traumatic Spinal Cord Dysfunction, type:	checkbox<br>1 impair_nt_scd___1 04.110 Paraplegia, Unspecified<br>2 impair_nt_scd___2 04.111 Paraplegia, Incomplete<br>3 impair_nt_scd___3 04.112 Paraplegia, Complete<br>4 impair_nt_scd___4 04.120 Quadriplegia, Unspecified<br>5 impair_nt_scd___5 04.1211 Quadriplegia, Incomplete C1-4<br>6 impair_nt_scd___6 04.1212 Quadriplegia, Incomplete C5-8<br>7 impair_nt_scd___7 04.1221 Quadriplegia, Complete C1-4<br>8 impair_nt_scd___8 04.1222 Quadriplegia, Complete C5-8<br>9 impair_nt_scd___9 04.130 Other Non-traumatic Spinal Cord Dysfunction
	77	[unk_36]<br>Show the field ONLY if:<br>[impair(4)] = '1'	If the above question cannot be answered, please check the box here:	checkbox<br>1 unk_36___1 Unknown
	78	[impair_t_scd]<br>Show the field ONLY if:<br>[impair(5)] = '1'	Traumatic Spinal Cord Dysfunction, type:	checkbox<br>1 impair_t_scd___1 04.210 Paraplegia, Unspecified<br>2 impair_t_scd___2 04.211 Paraplegia, Incomplete<br>3 impair_t_scd___3 04.212 Paraplegia, Complete<br>4 impair_t_scd___4 04.220 Quadriplegia, Unspecified<br>5 impair_t_scd___5 04.2211 Quadriplegia, Incomplete C1-4<br>6 impair_t_scd___6 04.2212 Quadriplegia, Incomplete C5-8<br>7 impair_t_scd___7 04.2221 Quadriplegia, Complete C1-4<br>8 impair_t_scd___8 04.2222 Quadriplegia, Complete C5-8<br>9 impair_t_scd___9 04.230 Other Traumatic Spinal Cord Dysfunction
	79	[unk_37]<br>Show the field ONLY if:<br>[impair(5)] = '1'	If the above question cannot be answered, please check the box here:	checkbox<br>1 unk_37___1 Unknown

	80	[impair_ampu]<br>Show the field ONLY if:<br>[impair(6)] = '1'	Amputation, type:	checkbox<br>1 impair_ampu___1 05.1 Unilateral Upper Limb Above the Elbow (AE)<br>2 impair_ampu___2 05.2 Unilateral Upper Limb Below the Elbow (BE)<br>3 impair_ampu___3 05.3 Unilateral Lower Limb Above the Knee (AK)<br>4 impair_ampu___4 05.4 Unilateral Lower Limb Below the Knee (BK)<br>5 impair_ampu___5 05.5 Bilateral Lower Limb Above the Knee (AK/AK<br>6 impair_ampu___6 05.6 Bilateral Lower Limb Above/Below Knee (AK/BK)<br>7 impair_ampu___7 05.7 Bilateral Lower Limb Below the Knee (BK/BK)<br>8 impair_ampu___8 05.9 Other Amputation
	81	[unk_38]<br>Show the field ONLY if:<br>[impair(6)] = '1'	If the above question cannot be answered, please check the box here:	checkbox<br>1 unk_38___1 Unknown
	82	[impair_arth]<br>Show the field ONLY if:<br>[impair(7)] = '1'	Arthritis, type:	checkbox<br>1 impair_arth___1 06.1 Rheumatoid Arthritis<br>2 impair_arth___2 06.2 Osteoarthritis<br>3 impair_arth___3 06.9 Other Arthritis
	83	[unk_39]<br>Show the field ONLY if:<br>[impair(7)] = '1'	If the above question cannot be answered, please check the box here:	checkbox<br>1 unk_39___1 Unknown
	84	[impair_pain]<br>Show the field ONLY if:<br>[impair(8)] = '1'	Pain Sydrome, type:	checkbox<br>1 impair_pain___1 07.1 Neck Pain<br>2 impair_pain___2 07.2 Back Pain<br>3 impair_pain___3 07.3 Exptremity Pain<br>4 impair_pain___4 07.9 Other Pain
	85	[unk_40]<br>Show the field ONLY if:<br>[impair(8)] = '1'	If the above question cannot be answered, please check the box here:	checkbox<br>1 unk_40___1 Unknown
	86	[impair_ortho]<br>Show the field ONLY if:<br>[impair(9)] = '1'	Orthopedic Disorder, type:	checkbox<br>1 impair_ortho___1 08.11 Unilateral Hip Fracture<br>2 impair_ortho___2 08.12 Bilateral Hip Fracture<br>3 impair_ortho___3 08.2 Femur (Shaft) Fracture<br>4 impair_ortho___4 08.3 Pelvic Fracture<br>5 impair_ortho___5 08.4 Major Multiple Fractures<br>6 impair_ortho___6 08.51 Unilateral Hip Replacement<br>7 impair_ortho___7 08.52 Bilateral Hip Replacements<br>8 impair_ortho___8 08.61 Unilateral Knee Replacement<br>9 impair_ortho___9 08.62 Bilateral Knee Replacements<br>10 impair_ortho___10 08.71 Knee and Hip Replacements (same side)<br>11 impair_ortho___11 08.72 Knee and Hip replacements (different sides)<br>12 impair_ortho___12 08.9 Other Orthopedic

87	[unk_41]<br><br>Show the field ONLY if:<br>[impair(9)] = '1'	If the above question cannot be answered, please check the box here:	checkbox<br>1 unk_41___1 Unknown
88	[impair_pulm]<br><br>Show the field ONLY if:<br>[impair(11)] = '1'	Pulmonary Disorder, type:	checkbox<br>1 impair_pulm___1 10.1 Chronic Obstructive Pulmonary Disease<br>2 impair_pulm___2 10.9 Other Pulmonary
89	[unk_42]<br><br>Show the field ONLY if:<br>[impair(11)] = '1'	If the above question cannot be answered, please check the box here:	checkbox<br>1 unk_42___1 Unknown
90	[impair_cong]<br><br>Show the field ONLY if:<br>[impair(13)] = '1'	Congenital Deformities, type:	checkbox<br>1 impair_cong___1 12.1 Spina Bifida<br>2 impair_cong___2 12.9 Other Congenital Deformity
91	[unk_43]<br><br>Show the field ONLY if:<br>[impair(13)] = '1'	If the above question cannot be answered, please check the box here:	checkbox<br>1 unk_43___1 Unknown
92	[impair_mmt]<br><br>Show the field ONLY if:<br>[impair(15)] = '1'	Major Multiple Trauma, type:	checkbox<br>1 impair_mmt___1 14.1 Brain + Spinal Cord Injury<br>2 impair_mmt___2 14.2 Brain + Multiple Fracture/Amputation<br>3 impair_mmt___3 14.3 Spinal Cord + Multiple Fracture/Amputation<br>4 impair_mmt___4 14.9 Other Multiple Trauma
93	[unk_44]<br><br>Show the field ONLY if:<br>[impair(15)] = '1'	If the above question cannot be answered, please check the box here:	checkbox<br>1 unk_44___1 Unknown
94	[impair_complex]<br><br>Show the field ONLY if:<br>[impair(18)] = '1'	Medically Complex, type:	checkbox<br>1 impair_complex___1 17.1 Infections<br>2 impair_complex___2 17.2 Neoplasms<br>3 impair_complex___3 17.31 Nutrition with Intubation/Parenteral Nutrition<br>4 impair_complex___4 17.32 Nutrition without Intubation/Parenteral Nutrition<br>5 impair_complex___5 17.4 Circulatory Disorders<br>6 impair_complex___6 17.51 Respiratory Disorders - Ventilator Dependent<br>7 impair_complex___7 17.52 Respiratory Disorders - Non-ventilator Dependent<br>8 impair_complex___8 17.6 Terminal Care<br>9 impair_complex___9 17.7 Skin Disorders<br>10 impair_complex___10 17.8 Medical/Surgical Complications<br>11 impair_complex___11 17.9 Other Medically Complex Conditions
95	[unk_45]<br><br>Show the field ONLY if:<br>[impair(18)] = '1'	If the above question cannot be answered, please check the box here:	checkbox<br>1 unk_45___1 Unknown
96	[dysph]	Dysphagia upon Acute Rehab admission?	yesno<br>1 Yes<br>0 No

	97	[unk_46]	If the above question cannot be answered, please check the box here:	checkbox<br>1 unk_46___1 Unknown
	98	[dysph_diet_adm]<br>Show the field ONLY if:<br>[dysph] = '1'	Indicate the most severe dysphagia diet (solid foods) administered during acute rehab admission:	dropdown<br>1 Regular<br>2 Dysphagia 3- Soft cut up<br>3 Dysphagia 2- mechanical soft<br>4 Dysphagia 1- Pureed<br>5 NPO
	99	[unk_47]<br>Show the field ONLY if:<br>[dysph] = '1'	If the above question cannot be answered, please check the box here:	checkbox<br>1 unk_47___1 Unknown
	100	[dysph_liqdiet_adm]<br>Show the field ONLY if:<br>[dysph] = '1'	Indicate the most severe dysphagia diet (liquids) administered during acute rehab admission:	dropdown<br>1 Thin<br>2 Nectar<br>3 Honey<br>4 No Liquids<br>5 No restriction, normal liquid diet
	101	[unk_48]<br>Show the field ONLY if:<br>[dysph] = '1'	If the above question cannot be answered, please check the box here:	checkbox<br>1 unk_48___1 Unknown
	102	[dysph_diet_disch]<br>Show the field ONLY if:<br>[dysph] = '1'	Indicate the most severe dysphagia diet (solid foods) administered at discharge from acute rehab:	dropdown<br>1 Regular<br>2 Dysphagia 3- Soft cut up<br>3 Dysphagia 2- mechanical soft<br>4 Dysphagia 1- Pureed<br>5 NPO
	103	[unk_49]<br>Show the field ONLY if:<br>[dysph] = '1'	If the above question cannot be answered, please check the box here:	checkbox<br>1 unk_49___1 Unknown
	104	[dysph_liqdiet_disch]<br>Show the field ONLY if:<br>[dysph] = '1'	Indicate the most severe dysphagia diet (liquids) administered at discharge from acute rehab:	dropdown<br>1 Thin<br>2 Nectar<br>3 Honey<br>4 No Liquids<br>5 No restriction, normal liquid diet
	105	[unk_50]<br>Show the field ONLY if:<br>[dysph] = '1'	If the above question cannot be answered, please check the box here:	checkbox<br>1 unk_50___1 Unknown
	106	[dysph_tube]<br>Show the field ONLY if:<br>[dysph] = '1'	If 'Yes' to dysphagia, was a percutaneous feeding tube placed?	yesno<br>1 Yes<br>0 No
	107	[unk_51]<br>Show the field ONLY if:<br>[dysph] = '1'	If the above question cannot be answered, please check the box here:	checkbox<br>1 unk_51___1 Unknown
	108	[second]	Secondary diagnosis?	checkbox<br>1 second___1 Neurological<br>2 second___2 Vascular<br>3 second___3 Pulmonary
	109	[unk_52]	If the above question cannot be answered, please check the box here:	checkbox<br>1 unk_52___1 Unknown

	110	[second_neuro]<br>Show the field ONLY if:<br>[second(1)] = '1'	Type of neurological condition:	checkbox<br>1 second_neuro___1 CVA<br>2 second_neuro___2 AIDP<br>3 second_neuro___3 Peripheral neuropathy<br>4 second_neuro___4 Plexopathy<br>5 second_neuro___5 Entrapment neuropathy<br>6 second_neuro___6 Myopathy<br>7 second_neuro___7 Encephalopathy
	111	[unk_53]<br>Show the field ONLY if:<br>[second(1)] = '1'	If the above question cannot be answered, please check the box here:	checkbox<br>1 unk_53___1 Unknown
	112	[second_vasc]<br>Show the field ONLY if:<br>[second(2)] = '1'	Type of vascular condition:	checkbox<br>1 second_vasc___1 DVT<br>2 second_vasc___2 Amputation
	113	[unk_54]<br>Show the field ONLY if:<br>[second(2)] = '1'	If the above question cannot be answered, please check the box here:	checkbox<br>1 unk_54___1 Unknown
	114	[second_pulm]<br>Show the field ONLY if:<br>[second(3)] = '1'	Type of pulmonary condition during acute rehab stay:	checkbox<br>1 second_pulm___1 Receiving oxygen at admission<br>2 second_pulm___2 Receiving oxygen at discharge
	115	[unk_55]<br>Show the field ONLY if:<br>[second(3)] = '1'	If the above question cannot be answered, please check the box here:	checkbox<br>1 unk_55___1 Unknown
	116	[comorbid]	Indicate any comorbid conditions upon admission to acute rehab:	checkbox<br>1 comorbid___1 Hypertension<br>2 comorbid___2 Obesity<br>3 comorbid___3 Diabetes mellitus<br>4 comorbid___4 Hypercholesterolemia<br>5 comorbid___5 Hyperlipidemia<br>6 comorbid___6 PVD<br>7 comorbid___7 CAD<br>8 comorbid___8 Hx of brain injury<br>9 comorbid___9 Hx of SCI<br>10 comorbid___10 Hx of respiratory disease<br>11 comorbid___11 HIV positive<br>12 comorbid___12 CP or developmental disability<br>13 comorbid___13 No comorbid conditions
	117	[unk_56]	If the above question cannot be answered, please check the box here:	checkbox<br>1 unk_56___1 Unknown
	118	[gg_tot_adm]	Total GG score at admission to acute rehab:	text (number)
	119	[unk_57]	If the above question cannot be answered, please check the box here:	checkbox<br>1 unk_57___1 Unknown
	120	[gg_tot_disch]	Total GG score at discharge from acute rehab:	text (number)
	121	[unk_58]	If the above question cannot be answered, please check the box here:	checkbox<br>1 unk_58___1 Unknown
	122	[change_gg_tot]	Change in Total GG (Discharge - Admission):	calc<br>Calculation: [gg_tot_disch] - [gg_tot_adm]
	123	[gg_cog_adm]	BIM score at admission to acute rehab:	text (number)
	124	[unk_59]	If the above question cannot be answered, please check the box here:	checkbox<br>1 unk_59___1 Unknown
	125	[gg_mot_adm]	Mobility GG score at admission to acute rehab:	text (number)

	126	[unk_60]	If the above question cannot be answered, please check the box here:	checkbox<br>1 | unk_60___1 | Unknown
	127	[gg_mot_disch]	Mobility GG score at discharge from acute rehab:	text (number)
	128	[unk_61]	If the above question cannot be answered, please check the box here:	checkbox<br>1 | unk_61___1 | Unknown
	129	[change_gg_mot]	Change in Mobility GG (Discharge - Admission):	calc<br>Calculation: [gg_mot_disch] - [gg_mot_adm]
	130	[skin_incidence]	Pressure wounds, number of locations affected during acute rehab admission: If more than 4 pressure wounds, report 4 most severe	dropdown<br>1 | 0<br>2 | 1<br>3 | 2<br>4 | 3<br>5 | 4
	131	[unk_62]	If the above question cannot be answered, please check the box here:	checkbox<br>1 | unk_62___1 | Unknown
	132	[skin_stage_1]<br>Show the field ONLY if:<br>[skin_incidence] = '2' or [skin_incidence] = '3' or [skin_incidence] = '4' or [skin_incidence] = '5'	Pressure wounds, wound stage of the first location:	dropdown<br>1 | 1<br>2 | 2<br>3 | 3<br>4 | 4<br>5 | Unstageable
	133	[unk_63]<br>Show the field ONLY if:<br>[skin_incidence] = '2' or [skin_incidence] = '3' or [skin_incidence] = '4' or [skin_incidence] = '5'	If the above question cannot be answered, please check the box here:	checkbox<br>1 | unk_63___1 | Unknown
	134	[skin_stage_2]<br>Show the field ONLY if:<br>[skin_incidence] = '5' or [skin_incidence] = '4' or [skin_incidence] = '3'	Pressure wounds, wound stage of the second location:	dropdown<br>1 | 1<br>2 | 2<br>3 | 3<br>4 | 4<br>5 | Unstageable
	135	[unk_64]<br>Show the field ONLY if:<br>[skin_incidence] = '5' or [skin_incidence] = '4' or [skin_incidence] = '3'	If the above question cannot be answered, please check the box here:	checkbox<br>1 | unk_64___1 | Unknown
	136	[skin_stage_3]<br>Show the field ONLY if:<br>[skin_incidence] = '5' or [skin_incidence] = '4'	Pressure wounds, wound stage of the third location:	dropdown<br>1 | 1<br>2 | 2<br>3 | 3<br>4 | 4<br>5 | Unstageable
	137	[unk_65]<br>Show the field ONLY if:<br>[skin_incidence] = '5' or [skin_incidence] = '4'	If the above question cannot be answered, please check the box here:	checkbox<br>1 | unk_65___1 | Unknown

	138	[skin_stage_4]<br><br>Show the field ONLY if:<br>[skin_incidence] = '5'	Pressure wounds, wound stage of the fourth location:	dropdown<br>1 | 1<br>2 | 2<br>3 | 3<br>4 | 4<br>5 | Unstageable
	139	[unk_66]<br><br>Show the field ONLY if:<br>[skin_incidence] = '5'	If the above question cannot be answered, please check the box here:	checkbox<br>1 | unk_66___1 | Unknown
	140	[skin_location_1]<br><br>Show the field ONLY if:<br>[skin_incidence] = '2' or [skin_incidence] = '3' or [skin_incidence] = '4' or [skin_incidence] = '5'	Pressure wounds, location of the first wound:	dropdown<br>1 | Face<br>2 | Occiput<br>3 | Ear (Left)<br>4 | Ear (Right)<br>5 | Shoulder (Left)<br>6 | Shoulder (Right)<br>7 | Cervical spine<br>8 | Elbow (Left)<br>9 | Elbow (Right)<br>10 | Thoracic spine<br>11 | Sternum<br>12 | Anterior hips (Left)<br>13 | Anterior hips (Right)<br>14 | Low back/PSIS (Left)<br>15 | Low back/PSIS (Right)<br>16 | Ischium (Left)<br>17 | Ischium (Right)<br>18 | Buttock (Left)<br>19 | Buttock (Right)<br>20 | Sacrum<br>21 | Coccyx<br>22 | Knee (Left)<br>23 | Knee (Right)<br>24 | Leg (Left)<br>25 | Leg (Right)<br>26 | Heel (Left)<br>27 | Heel (Right)<br>28 | Medial malleolus (Left)<br>29 | Medial malleolus (Right)<br>30 | Lateral malleolus (Left)<br>31 | Lateral malleolus (Right)<br>32 | Foot (Left)<br>33 | Foot (Right)<br>34 | Toes (Left)<br>35 | Toes (Right)
	141	[unk_67]<br><br>Show the field ONLY if:<br>[skin_incidence] = '2' or [skin_incidence] = '3' or [skin_incidence] = '4' or [skin_incidence] = '5'	If the above question cannot be answered, please check the box here:	checkbox<br>1 | unk_67___1 | Unknown

	142	[skin_location_2]<br><br>Show the field ONLY if:<br>[skin_incidence] = '3' or [skin_incidence] = '4' or [skin_incidence] = '5'	Pressure wounds, location of the second wound:	dropdown<br>1 Face<br>2 Occiput<br>3 Ear (Left)<br>4 Ear (Right)<br>5 Shoulder (Left)<br>6 Shoulder (Right)<br>7 Cervical spine<br>8 Elbow (Left)<br>9 Elbow (Right)<br>10 Thoracic spine<br>11 Sternum<br>12 Anterior hips (Left)<br>13 Anterior hips (Right)<br>14 Low back/PSIS (Left)<br>15 Low back/PSIS (Right)<br>16 Ischium (Left)<br>17 Ischium (Right)<br>18 Buttock (Left)<br>19 Buttock (Right)<br>20 Sacrum<br>21 Coccyx<br>22 Knee (Left)<br>23 Knee (Right)<br>24 Leg (Left)<br>25 Leg (Right)<br>26 Heel (Left)<br>27 Heel (Right)<br>28 Medial malleolus (Left)<br>29 Medial malleolus (Right)<br>30 Lateral malleolus (Left)<br>31 Lateral malleolus (Right)<br>32 Foot (Left)<br>33 Foot (Right)<br>34 Toes (Left)<br>35 Toes (Right)
	143	[unk_68]<br><br>Show the field ONLY if:<br>[skin_incidence] = '3' or [skin_incidence] = '4' or [skin_incidence] = '5'	If the above question cannot be answered, please check the box here:	checkbox<br>1 unk_68___1 Unknown

	144	[skin_location_3]<br><br>Show the field ONLY if:<br>[skin_incidence] = '4' or [skin_incidence] = '5'	Pressure wounds, location of the third wound:	dropdown<br>1 Face<br>2 Occiput<br>3 Ear (Left)<br>4 Ear (Right)<br>5 Shoulder (Left)<br>6 Shoulder (Right)<br>7 Cervical spine<br>8 Elbow (Left)<br>9 Elbow (Right)<br>10 Thoracic spine<br>11 Sternum<br>12 Anterior hips (Left)<br>13 Anterior hips (Right)<br>14 Low back/PSIS (Left)<br>15 Low back/PSIS (Right)<br>16 Ischium (Left)<br>17 Ischium (Right)<br>18 Buttock (Left)<br>19 Buttock (Right)<br>20 Sacrum<br>21 Coccyx<br>22 Knee (Left)<br>23 Knee (Right)<br>24 Leg (Left)<br>25 Leg (Right)<br>26 Heel (Left)<br>27 Heel (Right)<br>28 Medial malleolus (Left)<br>29 Medial malleolus (Right)<br>30 Lateral malleolus (Left)<br>31 Lateral malleolus (Right)<br>32 Foot (Left)<br>33 Foot (Right)<br>34 Toes (Left)<br>35 Toes (Right)
	145	[unk_69]<br><br>Show the field ONLY if:<br>[skin_incidence] = '4' or [skin_incidence] = '5'	If the above question cannot be answered, please check the box here:	checkbox<br>1 unk_69___1 Unknown

	146	[skin_location_4]<br>Show the field ONLY if:<br>[skin_incidence] = '5'	Pressure wounds, location of the fourth wound:	dropdown<br>1 Face<br>2 Occiput<br>3 Ear (Left)<br>4 Ear (Right)<br>5 Shoulder (Left)<br>6 Shoulder (Right)<br>7 Cervical spine<br>8 Elbow (Left)<br>9 Elbow (Right)<br>10 Thoracic spine<br>11 Sternum<br>12 Anterior hips (Left)<br>13 Anterior hips (Right)<br>14 Low back/PSIS (Left)<br>15 Low back/PSIS (Right)<br>16 Ischium (Left)<br>17 Ischium (Right)<br>18 Buttock (Left)<br>19 Buttock (Right)<br>20 Sacrum<br>21 Coccyx<br>22 Knee (Left)<br>23 Knee (Right)<br>24 Leg (Left)<br>25 Leg (Right)<br>26 Heel (Left)<br>27 Heel (Right)<br>28 Medial malleolus (Left)<br>29 Medial malleolus (Right)<br>30 Lateral malleolus (Left)<br>31 Lateral malleolus (Right)<br>32 Foot (Left)<br>33 Foot (Right)<br>34 Toes (Left)<br>35 Toes (Right)
	147	[unk_70]<br>Show the field ONLY if:<br>[skin_incidence] = '5'	If the above question cannot be answered, please check the box here:	checkbox<br>1 unk_70___1 Unknown
	148	[care]	Was this patient transferred from acute rehab to acute care?	yesno<br>1 Yes<br>0 No
	149	[unk_71]	If the above question cannot be answered, please check the box here:	checkbox<br>1 unk_71___1 Unknown
	150	[los_care]<br>Show the field ONLY if:<br>[care] = '1'	Length of stay in acute care after transfer:<br>*Indicate the number of days stayed*	text (number)
	151	[unk_72]<br>Show the field ONLY if:<br>[care] = '1'	If the above question cannot be answered, please check the box here:	checkbox<br>1 unk_72___1 Unknown

	152	[acute_reason]<br>Show the field ONLY if:<br>[care] = '1'	Please indicate the reason for transfer to acute care from acute rehab:	checkbox<br>1 acute_reason___1 Respiratory Distress<br>2 acute_reason___2 Sepsis<br>3 acute_reason___3 Cardiac Distress<br>4 acute_reason___4 AMS<br>5 acute_reason___5 Bleeding
	153	[unk_73]<br>Show the field ONLY if:<br>[care] = '1'	If the above question cannot be answered, please check the box here:	checkbox<br>1 unk_73___1 Unknown
	154	[confirm]<br>Show the field ONLY if:<br>[care] = '1'	You have indicated that this patient was transferred from Acute Rehab to Acute Care. In the event that this patient then returned to Acute Rehab, we must capture the discharge GG scores from their FINAL discharge from Acute Rehab. By checking 'Yes' below, you verify that the data you have entered under GG scores at discharge from Acute Rehab (total, motor, and cognitive if appropriate) reflect those scores from the final Acute Rehab discharge. If you are unsure, please verify before checking 'Yes' and concluding data entry.	yesno<br>1 Yes<br>0 No
	155	[disp]	Please indicate the final discharge disposition from the acute rehab unit:	dropdown<br>1 Home<br>2 Acute Hospital<br>3 SAR<br>4 LTAC<br>5 Hospice<br>6 Deceased
	156	[unk_74]	If the above question cannot be answered, please check the box here:	checkbox<br>1 unk_74___1 Unknown
	157	[o2]<br>Show the field ONLY if:<br>[disp] = '1'	Was the patient discharged from acute rehab to home on oxygen?	yesno<br>1 Yes<br>0 No
	158	[unk_75]<br>Show the field ONLY if:<br>[disp] = '1'	If the above question cannot be answered, please check the box here:	checkbox<br>1 unk_75___1 Unknown
	159	[o2_lpm]<br>Show the field ONLY if:<br>[o2] = '1'	Indicate the flow rate (lpm) administered at time of discharge from acute rehab:<br>*liters per minute*	dropdown<br>1 1 lpm<br>2 2 lpm<br>3 3 lpm<br>4 4 lpm
	160	[unk_76]<br>Show the field ONLY if:<br>[o2] = '1'	If the above question cannot be answered, please check the box here:	checkbox<br>1 unk_76___1 Unknown
	161	[retrospective_data_complete]	Section Header: *Form Status*<br>Complete?	dropdown<br>0 Incomplete<br>1 Unverified<br>2 Complete
Instrument: **Cognitive Measures** (cognitive_measures)				
	162	[moca_file]	MOCA Upload:	file
	163	[moca_vis]	MOCA, Visuospatial/Executive:<br>*# of points out of 5*	text (number)
	164	[moca_name]	MOCA, Naming:<br>*# of points out of 3*	text (number)
	165	[moca_att]	MOCA, Attention:<br>*# of points out of 6*	text (number)
	166	[moca_lang]	MOCA, Language:<br>*# of points out of 3*	text (number)

	167	[moca_abs]	MOCA, Abstraction:<br>*# of points out of 2*	text (number)
	168	[moca_dr]	MOCA, Delayed Recall:<br>*# of points out of 5*	text (number)
	169	[moca_ori]	MOCA, Orientation:<br>*# of points out of 6*	text (number)
	170	[moca_total]	MOCA, Total Score:<br>*# of points out of 30, use uncorrected score (not +1 for education)*	text (number)
	171	[gad7_file]	GAD-7 Upload:	file
	172	[gad7_1]	Section Header: *GAD-7*<br>Question 1	radio (Matrix)<br>1 0<br>2 1<br>3 2<br>4 3
	173	[gad7_2]	Question 2	radio (Matrix)<br>1 0<br>2 1<br>3 2<br>4 3
	174	[gad7_3]	Question 3	radio (Matrix)<br>1 0<br>2 1<br>3 2<br>4 3
	175	[gad7_4]	Question 4	radio (Matrix)<br>1 0<br>2 1<br>3 2<br>4 3
	176	[gad7_5]	Question 5	radio (Matrix)<br>1 0<br>2 1<br>3 2<br>4 3
	177	[gad7_6]	Question 6	radio (Matrix)<br>1 0<br>2 1<br>3 2<br>4 3
	178	[gad7_7]	Question 7	radio (Matrix)<br>1 0<br>2 1<br>3 2<br>4 3
	179	[gad7_tot]	GAD-7, Total Score:	text (number)
	180	[gad7_diff]	GAD-7, If you checked off any problems, how difficult have these made it for you to do your work, take care of things at home, or get along with other people?	radio<br>1 Not difficult at all<br>2 Somewhat difficult<br>3 Very difficult<br>4 Extremely difficult

	181	[gad7_anx]	GAD-7, Interpretation?	radio<br>0 Score less than 5, Minimal anxiety<br>1 Score of 5-9, Mild anxiety<br>2 Score of 10-14, Moderate anxiety<br>3 Score of 15 or higher, Severe anxiety
	182	[phq9_file]	PHQ-9 Upload:	file
	183	[phq_1]	Section Header: *PHQ-9*<br>Question 1	radio (Matrix)<br>1 0<br>2 1<br>3 2<br>4 3
	184	[phq_2]	Question 2	radio (Matrix)<br>1 0<br>2 1<br>3 2<br>4 3
	185	[phq_3]	Question 3	radio (Matrix)<br>1 0<br>2 1<br>3 2<br>4 3
	186	[phq_4]	Question 4	radio (Matrix)<br>1 0<br>2 1<br>3 2<br>4 3
	187	[phq_5]	Question 5	radio (Matrix)<br>1 0<br>2 1<br>3 2<br>4 3
	188	[phq_6]	Question 6	radio (Matrix)<br>1 0<br>2 1<br>3 2<br>4 3
	189	[phq_7]	Question 7	radio (Matrix)<br>1 0<br>2 1<br>3 2<br>4 3
	190	[phq_8]	Question 8	radio (Matrix)<br>1 0<br>2 1<br>3 2<br>4 3

	191	[phq_9]	Question 9	radio (Matrix)<br>1 0<br>2 1<br>3 2<br>4 3
	192	[phq_tot]	PHQ-9, Total Score:	text (number)
	193	[phq_diff]	PHQ-9, If you checked off any problems, how difficult have these problems made it for you to do your work, take care of things at home, or get along with other people?	radio<br>1 Not difficult at all<br>2 Somewhat difficult<br>3 Very difficult<br>4 Extremely difficult
	194	[phq_int]	PHQ-9 Interpretation:	radio<br>0 1-4, Minimal depression<br>1 5-9, Mild depression<br>2 10-14, Moderate depression<br>3 15-19, Moderately severe depression<br>4 20-27, Severe depression
	195	[speech]	Was this patient administered or recommended for speech therapy?	yesno<br>1 Yes<br>0 No
	196	[fim_file]	FIM Upload:	file
	197	[fim_comp]	Section Header: *FIM, Communication and Social Cognition*<br>Comprehension	radio (Matrix)<br>1 7 - Complete Independence<br>2 6 - Modified Independence<br>3 5 - Supervision<br>4 4 - Minimal Prompting<br>5 3 - Moderate Prompting<br>6 2 - Maximal Prompting<br>7 1 - Total Assistance
	198	[fim_exp]	Expression	radio (Matrix)<br>1 7 - Complete Independence<br>2 6 - Modified Independence<br>3 5 - Supervision<br>4 4 - Minimal Prompting<br>5 3 - Moderate Prompting<br>6 2 - Maximal Prompting<br>7 1 - Total Assistance
	199	[fim_soc]	Social Interaction	radio (Matrix)<br>1 7 - Complete Independence<br>2 6 - Modified Independence<br>3 5 - Supervision<br>4 4 - Minimal Prompting<br>5 3 - Moderate Prompting<br>6 2 - Maximal Prompting<br>7 1 - Total Assistance

200	[fim_prob]	Problem Solving	radio (Matrix)<br>1 7 - Complete Independence<br>2 6 - Modified Independence<br>3 5 - Supervision<br>4 4 - Minimal Prompting<br>5 3 - Moderate Prompting<br>6 2 - Maximal Prompting<br>7 1 - Total Assistance
201	[fim_mem]	Memory	radio (Matrix)<br>1 7 - Complete Independence<br>2 6 - Modified Independence<br>3 5 - Supervision<br>4 4 - Minimal Prompting<br>5 3 - Moderate Prompting<br>6 2 - Maximal Prompting<br>7 1 - Total Assistance
202	[clqt_file]	CLQT Upload:	file
203	[clqt_att]	Section Header: *CLQT*<br>Attention	radio (Matrix)<br>1 Within Normal Limits (WNL, 4)<br>2 Mild (3)<br>3 Moderate (2)<br>4 Severe (1)
204	[clqt_mem]	Memory	radio (Matrix)<br>1 Within Normal Limits (WNL, 4)<br>2 Mild (3)<br>3 Moderate (2)<br>4 Severe (1)
205	[clqt_exec]	Executive Functions	radio (Matrix)<br>1 Within Normal Limits (WNL, 4)<br>2 Mild (3)<br>3 Moderate (2)<br>4 Severe (1)
206	[clqt_lang]	Language	radio (Matrix)<br>1 Within Normal Limits (WNL, 4)<br>2 Mild (3)<br>3 Moderate (2)<br>4 Severe (1)
207	[clqt_vis]	Visuospatial Skills	radio (Matrix)<br>1 Within Normal Limits (WNL, 4)<br>2 Mild (3)<br>3 Moderate (2)<br>4 Severe (1)
208	[clqt_clock]	Clock Drawing	radio (Matrix)<br>1 Within Normal Limits (WNL, 4)<br>2 Mild (3)<br>3 Moderate (2)<br>4 Severe (1)
209	[att_score]	CLQT Attention Score: For patients age < 70, WNL (215-180), Mild (179-125), Mod (124-50), Severe (49-0); patients age >= 70, WNL (215-160), Mild (159-100), Mod (99-40), Severe (39-0)	text (number)

	210	[mem_score]	CLQT Memory Score: For patients age < 70, WNL (185-155), Mild (154-141), Mod (140-110), Severe (109-0); patients age >= 70, WNL (185-141), Mild (140-115), Mod (114-80), Severe (79-0)	text (number)
	211	[exec_score]	CLQT Executive Function Score: For patients age < 70, WNL (40-24), Mild (23-20), Mod (19-16), Severe (15-0); patients age >= 70, WNL (40-19), Mild (18-14), Mod (13-8), Severe (7-0)	text (number)
	212	[lang_score]	CLQT Language Score: For patients age < 70, WNL (37-29), Mild (28-25), Mod (24-21), Severe (20-0); patients age >= 70, WNL (37-28), Mild (27-25), Mod (24-16), Severe (15-0)	text (number)
	213	[vis_score]	CLQT Visuospatial Skills Score: For patients age < 70, WNL (105-82), Mild (81-52), Mod (51-42), Severe (41-0); patients age >= 70, WNL (105-62), Mild (61-37), Mod (36-22), Severe (21-0)	text (number)
	214	[clock_score]	CLQT Clock Drawing Score: For patients age < 70, WNL (13-12), Mild (11-10), Mod (9-8), Severe (7-0); patients age >= 70, WNL (13-11), Mild (10-9), Mod (8-7), Severe (6-0)	text (number)
	215	[cognitive_measures_complete]	Section Header: *Form Status*<br>Complete?	dropdown<br>0 Incomplete<br>1 Unverified<br>2 Complete
**Instrument: Skin Project (skin_project)**				
	216	[los_sp]	Length of stay in acute care:<br>*Indicate the number of days stayed*	text (number)
	217	[unk_77]	If the above question cannot be answered, please check the box here:	checkbox<br>1 unk_77___1 Unknown
	218	[icu_sp]	Was this patient transferred to the ICU?	yesno<br>1 Yes<br>0 No
	219	[unk_78]	If the above question cannot be answered, please check the box here:	checkbox<br>1 unk_78___1 Unknown
	220	[los_icu_sp]<br>Show the field ONLY if:<br>[icu] = '1'	Length of stay in the ICU:<br>*Indicate the number of days stayed*	text (number)
	221	[unk_79]<br>Show the field ONLY if:<br>[icu] = '1'	If the above question cannot be answered, please check the box here:	checkbox<br>1 unk_79___1 Unknown
	222	[steroid_sp]	Were systemic steroids administered?	yesno<br>1 Yes<br>0 No
	223	[unk_80]	If the above question cannot be answered, please check the box here:	checkbox<br>1 unk_80___1 Unknown
	224	[suppress_sp]	Were immunosuppressants (e.g. monoclonal antibody therapies) administered?	yesno<br>1 Yes<br>0 No
	225	[unk_81]	If the above question cannot be answered, please check the box here:	checkbox<br>1 unk_81___1 Unknown
	226	[mech_vent_sp]	Did the patient receive mechanical ventilation?	yesno<br>1 Yes<br>0 No
	227	[unk_82]	If the above question cannot be answered, please check the box here:	checkbox<br>1 unk_82___1 Unknown

	228	[trach_sp]	Did this patient receive a tracheostomy?	yesno<br>1 Yes<br>0 No
	229	[unk_83]	If the above question cannot be answered, please check the box here:	checkbox<br>1 unk_83___1 Unknown
	230	[prone_sp]	Did the patient experience proning?	yesno<br>1 Yes<br>0 No
	231	[unk_84]	If the above question cannot be answered, please check the box here:	checkbox<br>1 unk_84___1 Unknown
	232	[ecmo_sp]	Was the patient administered ECMO?	yesno<br>1 Yes<br>0 No
	233	[unk_85]	If the above question cannot be answered, please check the box here:	checkbox<br>1 unk_85___1 Unknown
	234	[plasma_sp]	Did this patient receive convalescent plasma?	yesno<br>1 Yes<br>0 No
	235	[unk_86]	If the above question cannot be answered, please check the box here:	checkbox<br>1 unk_86___1 Unknown
	236	[ferritin_sp]	Peak ferritin level (ng/mL) during their stay:	text (number)
	237	[unk_87]	If the above question cannot be answered, please check the box here:	checkbox<br>1 unk_87___1 Unknown
	238	[crp_sp]	Peak CRP level (mg/dL) during their stay:	text (number)
	239	[unk_88]	If the above question cannot be answered, please check the box here:	checkbox<br>1 unk_88___1 Unknown
	240	[ddimer_sp]	Peak D-dimer level (ng/mL) during their stay:	text (number)
	241	[unk_89]	If the above question cannot be answered, please check the box here:	checkbox<br>1 unk_89___1 Unknown
	242	[il_6_sp]	Peak IL-6 level (pg/mL) during their stay:	text (number)
	243	[unk_90]	If the above question cannot be answered, please check the box here:	checkbox<br>1 unk_90___1 Unknown
	244	[a1c_sp]	Peak HBA1C level (mmol/mol) during their stay:	text (number)
	245	[unk_91]	If the above question cannot be answered, please check the box here:	checkbox<br>1 unk_91___1 Unknown

	246	[impair_sp]	Please indicate the appropriate Impairment code and answer appropriate followup questions according to which codes apply:	checkbox<br>1 impair_sp___1 Stroke<br>2 impair_sp___2 Brain Dysfunction<br>3 impair_sp___3 Neurologic Condition<br>4 impair_sp___4 Non-traumatic Spinal Cord Dysfunction<br>5 impair_sp___5 Traumatic Spinal Cord Dysfunction<br>6 impair_sp___6 Amputation<br>7 impair_sp___7 Arthritis<br>8 impair_sp___8 Pain Syndrome<br>9 impair_sp___9 Orthopedic Disorder<br>10 impair_sp___10 Cardiac<br>11 impair_sp___11 Pulmonary Disorder<br>12 impair_sp___12 Burns<br>13 impair_sp___13 Congenital Deformities<br>14 impair_sp___14 Other Disabling Impairment<br>15 impair_sp___15 Major Multiple Trauma<br>16 impair_sp___16 Developmental Disability<br>17 impair_sp___17 Debility (Non-cardiac/Non-Pulmonary)<br>18 impair_sp___18 Medically Complex<br>19 impair_sp___19 No Impairment
	247	[unk_92]	If the above question cannot be answered, please check the box here:	checkbox<br>1 unk_92___1 Unknown
	248	[impair_stroke_sp]<br>Show the field ONLY if:<br>[impair(1)] = '1'	Stroke, type:	checkbox<br>1 impair_stroke_sp___1 01.1 Left Body Involvement (Right Brain)<br>2 impair_stroke_sp___2 01.2 Right Body Involvement (Left Brain)<br>3 impair_stroke_sp___3 01.3 Bilateral Involvement<br>4 impair_stroke_sp___4 01.4 No Paresis<br>5 impair_stroke_sp___5 01.9 Other Stroke
	249	[unk_93]<br>Show the field ONLY if:<br>[impair(1)] = '1'	If the above question cannot be answered, please check the box here:	checkbox<br>1 unk_93___1 Unknown
	250	[impair_brain_sp]<br>Show the field ONLY if:<br>[impair(2)] = '1'	Brain Dysfunction, type:	checkbox<br>1 impair_brain_sp___1 02.1 Non-traumatic<br>2 impair_brain_sp___2 02.21 Traumatic, Open Injury<br>3 impair_brain_sp___3 02.22 Traumatic, Closed Injury<br>4 impair_brain_sp___4 02.9 Other Brain
	251	[unk_94]<br>Show the field ONLY if:<br>[impair(2)] = '1'	If the above question cannot be answered, please check the box here:	checkbox<br>1 unk_94___1 Unknown

252	[impair_neuro_sp]<br>Show the field ONLY if:<br>[impair(3)] = '1'	Neurologic Condition, type:	checkbox<br>1 impair_neuro_sp___1 03.1 Multiple Sclerosis<br>2 impair_neuro_sp___2 03.2 Parkinsonism<br>3 impair_neuro_sp___3 03.3 Polyneuropathy<br>4 impair_neuro_sp___4 03.4 Guillain-Barre Syndrome<br>5 impair_neuro_sp___5 03.5 Cerebral Palsy<br>6 impair_neuro_sp___6 03.8 Neuromuscular Disorders<br>7 impair_neuro_sp___7 03.9 Other Neurologic
253	[unk_95]<br>Show the field ONLY if:<br>[impair(3)] = '1'	If the above question cannot be answered, please check the box here:	checkbox<br>1 unk_95___1 Unknown
254	[impair_nt_scd_sp]<br>Show the field ONLY if:<br>[impair(4)] = '1'	Non-traumatic Spinal Cord Dysfunction, type:	checkbox<br>1 impair_nt_scd_sp___1 04.110 Paraplegia, Unspecified<br>2 impair_nt_scd_sp___2 04.111 Paraplegia, Incomplete<br>3 impair_nt_scd_sp___3 04.112 Paraplegia, Complete<br>4 impair_nt_scd_sp___4 04.120 Quadriplegia, Unspecified<br>5 impair_nt_scd_sp___5 04.1211 Quadriplegia, Incomplete C1-4<br>6 impair_nt_scd_sp___6 04.1212 Quadriplegia, Incomplete C5-8<br>7 impair_nt_scd_sp___7 04.1221 Quadriplegia, Complete C1-4<br>8 impair_nt_scd_sp___8 04.1222 Quadriplegia, Complete C5-8<br>9 impair_nt_scd_sp___9 04.130 Other Non-traumatic Spinal Cord Dysfunction
255	[unk_96]<br>Show the field ONLY if:<br>[impair(4)] = '1'	If the above question cannot be answered, please check the box here:	checkbox<br>1 unk_96___1 Unknown
256	[impair_t_scd_sp]<br>Show the field ONLY if:<br>[impair(5)] = '1'	Traumatic Spinal Cord Dysfunction, type:	checkbox<br>1 impair_t_scd_sp___1 04.210 Paraplegia, Unspecified<br>2 impair_t_scd_sp___2 04.211 Paraplegia, Incomplete<br>3 impair_t_scd_sp___3 04.212 Paraplegia, Complete<br>4 impair_t_scd_sp___4 04.220 Quadriplegia, Unspecified<br>5 impair_t_scd_sp___5 04.2211 Quadriplegia, Incomplete C1-4<br>6 impair_t_scd_sp___6 04.2212 Quadriplegia, Incomplete C5-8<br>7 impair_t_scd_sp___7 04.2221 Quadriplegia, Complete C1-4<br>8 impair_t_scd_sp___8 04.2222 Quadriplegia, Complete C5-8<br>9 impair_t_scd_sp___9 04.230 Other Traumatic Spinal Cord Dysfunction
257	[unk_97]<br>Show the field ONLY if:<br>[impair(5)] = '1'	If the above question cannot be answered, please check the box here:	checkbox<br>1 unk_97___1 Unknown

258	[impair_ampu_sp]<br><br>Show the field ONLY if:<br>[impair(6)] = '1'	Amputation, type:	checkbox<br>1 impair_ampu_sp___1 05.1 Unilateral Upper Limb Above the Elbow (AE)<br>2 impair_ampu_sp___2 05.2 Unilateral Upper Limb Below the Elbow (BE)<br>3 impair_ampu_sp___3 05.3 Unilateral Lower Limb Above the Knee (AK)<br>4 impair_ampu_sp___4 05.4 Unilateral Lower Limb Below the Knee (BK)<br>5 impair_ampu_sp___5 05.5 Bilateral Lower Limb Above the Knee (AK/AK<br>6 impair_ampu_sp___6 05.6 Bilateral Lower Limb Above/Below Knee (AK/BK)<br>7 impair_ampu_sp___7 05.7 Bilateral Lower Limb Below the Knee (BK/BK)<br>8 impair_ampu_sp___8 05.9 Other Amputation
259	[unk_98]<br><br>Show the field ONLY if:<br>[impair(6)] = '1'	If the above question cannot be answered, please check the box here:	checkbox<br>1 unk_98___1 Unknown
260	[impair_arth_sp]<br><br>Show the field ONLY if:<br>[impair(7)] = '1'	Arthritis, type:	checkbox<br>1 impair_arth_sp___1 06.1 Rheumatoid Arthritis<br>2 impair_arth_sp___2 06.2 Osteoarthritis<br>3 impair_arth_sp___3 06.9 Other Arthritis
261	[unk_99]<br><br>Show the field ONLY if:<br>[impair(7)] = '1'	If the above question cannot be answered, please check the box here:	checkbox<br>1 unk_99___1 Unknown
262	[impair_pain_sp]<br><br>Show the field ONLY if:<br>[impair(8)] = '1'	Pain Sydrome, type:	checkbox<br>1 impair_pain_sp___1 07.1 Neck Pain<br>2 impair_pain_sp___2 07.2 Back Pain<br>3 impair_pain_sp___3 07.3 Exptremity Pain<br>4 impair_pain_sp___4 07.9 Other Pain
263	[unk_100]<br><br>Show the field ONLY if:<br>[impair(8)] = '1'	If the above question cannot be answered, please check the box here:	checkbox<br>1 unk_100___1 Unknown

	264	[impair_ortho_sp]<br><br>Show the field ONLY if:<br>[impair(9)] = '1'	Orthopedic Disorder, type:	checkbox<br>1 impair_ortho_sp___1 08.11 Unilateral Hip Fracture<br>2 impair_ortho_sp___2 08.12 Bilateral Hip Fracture<br>3 impair_ortho_sp___3 08.2 Femur (Shaft) Fracture<br>4 impair_ortho_sp___4 08.3 Pelvic Fracture<br>5 impair_ortho_sp___5 08.4 Major Multiple Fractures<br>6 impair_ortho_sp___6 08.51 Unilateral Hip Replacement<br>7 impair_ortho_sp___7 08.52 Bilateral Hip Replacements<br>8 impair_ortho_sp___8 08.61 Unilateral Knee Replacement<br>9 impair_ortho_sp___9 08.62 Bilateral Knee Replacements<br>10 impair_ortho_sp___10 08.71 Knee and Hip Replacements (same side)<br>11 impair_ortho_sp___11 08.72 Knee and Hip replacements (different sides)<br>12 impair_ortho_sp___12 08.9 Other Orthopedic
	265	[unk_101]<br><br>Show the field ONLY if:<br>[impair(9)] = '1'	If the above question cannot be answered, please check the box here:	checkbox<br>1 unk_101___1 Unknown
	266	[impair_pulm_sp]<br><br>Show the field ONLY if:<br>[impair(11)] = '1'	Pulmonary Disorder, type:	checkbox<br>1 impair_pulm_sp___1 10.1 Chronic Obstructive Pulmonary Disease<br>2 impair_pulm_sp___2 10.9 Other Pulmonary
	267	[unk_102]<br><br>Show the field ONLY if:<br>[impair(11)] = '1'	If the above question cannot be answered, please check the box here:	checkbox<br>1 unk_102___1 Unknown
	268	[impair_cog_sp]<br><br>Show the field ONLY if:<br>[impair(13)] = '1'	Congenital Deformities, type:	checkbox<br>1 impair_cog_sp___1 12.1 Spina Bifida<br>2 impair_cog_sp___2 12.9 Other Congenital Deformity
	269	[unk_103]<br><br>Show the field ONLY if:<br>[impair(13)] = '1'	If the above question cannot be answered, please check the box here:	checkbox<br>1 unk_103___1 Unknown
	270	[impair_mmt_sp]<br><br>Show the field ONLY if:<br>[impair(15)] = '1'	Major Multiple Trauma, type:	checkbox<br>1 impair_mmt_sp___1 14.1 Brain + Spinal Cord Injury<br>2 impair_mmt_sp___2 14.2 Brain + Multiple Fracture/Amputation<br>3 impair_mmt_sp___3 14.3 Spinal Cord + Multiple Fracture/Amputation<br>4 impair_mmt_sp___4 14.9 Other Multiple Trauma
	271	[unk_104]<br><br>Show the field ONLY if:<br>[impair(15)] = '1'	If the above question cannot be answered, please check the box here:	checkbox<br>1 unk_104___1 Unknown

	272	[impair_complex_sp]<br>Show the field ONLY if:<br>[impair(18)] = '1'	Medically Complex, type:	checkbox<br>1 impair_complex_sp___1 17.1 Infections<br>2 impair_complex_sp___2 17.2 Neoplasms<br>3 impair_complex_sp___3 17.31 Nutrition with Intubation/Parenteral Nutrition<br>4 impair_complex_sp___4 17.32 Nutrition without Intubation/Parenteral Nutrition<br>5 impair_complex_sp___5 17.4 Circulatory Disorders<br>6 impair_complex_sp___6 17.51 Respiratory Disorders - Ventilator Dependent<br>7 impair_complex_sp___7 17.52 Respiratory Disorders - Non-ventilator Dependent<br>8 impair_complex_sp___8 17.6 Terminal Care<br>9 impair_complex_sp___9 17.7 Skin Disorders<br>10 impair_complex_sp___10 17.8 Medical/Surgical Complications<br>11 impair_complex_sp___11 17.9 Other Medically Complex Conditions
	273	[unk_105]<br>Show the field ONLY if:<br>[impair(18)] = '1'	If the above question cannot be answered, please check the box here:	checkbox<br>1 unk_105___1 Unknown
	274	[second_sp]	Secondary diagnosis?	checkbox<br>1 second_sp___1 Neurological<br>2 second_sp___2 Vascular<br>3 second_sp___3 Pulmonary
	275	[unk_106]	If the above question cannot be answered, please check the box here:	checkbox<br>1 unk_106___1 Unknown
	276	[second_neuro_sp]<br>Show the field ONLY if:<br>[second(1)] = '1'	Type of neurological condition:	checkbox<br>1 second_neuro_sp___1 CVA<br>2 second_neuro_sp___2 AIDP<br>3 second_neuro_sp___3 Peripheral neuropathy<br>4 second_neuro_sp___4 Plexopathy<br>5 second_neuro_sp___5 Entrapment neuropathy<br>6 second_neuro_sp___6 Myopathy<br>7 second_neuro_sp___7 Encephalopathy
	277	[unk_107]<br>Show the field ONLY if:<br>[second(1)] = '1'	If the above question cannot be answered, please check the box here:	checkbox<br>1 unk_107___1 Unknown
	278	[second_vasc_sp]<br>Show the field ONLY if:<br>[second(2)] = '1'	Type of vascular condition:	checkbox<br>1 second_vasc_sp___1 DVT<br>2 second_vasc_sp___2 Amputation
	279	[unk_108]<br>Show the field ONLY if:<br>[second(2)] = '1'	If the above question cannot be answered, please check the box here:	checkbox<br>1 unk_108___1 Unknown
	280	[second_pulm_sp]<br>Show the field ONLY if:<br>[second(3)] = '1'	Type of pulmonary condition during acute rehab stay:	checkbox<br>1 second_pulm_sp___1 Receiving oxygen at admission<br>2 second_pulm_sp___2 Receiving oxygen at discharge

281	[unk_109]<br>Show the field ONLY if:<br>[second(3)] = '1'	If the above question cannot be answered, please check the box here:	checkbox<br>1 unk_109___1 Unknown
282	[comorbid_sp]	Indicate any comorbid conditions upon admission to acute rehab:	checkbox<br>1 comorbid_sp___1 Hypertension<br>2 comorbid_sp___2 Obesity<br>3 comorbid_sp___3 Diabetes mellitus<br>4 comorbid_sp___4 Hypercholesterolemia<br>5 comorbid_sp___5 Hyperlipidemia<br>6 comorbid_sp___6 PVD<br>7 comorbid_sp___7 CAD<br>8 comorbid_sp___8 Hx of brain injury<br>9 comorbid_sp___9 Hx of SCI<br>10 comorbid_sp___10 Hx of respiratory disease<br>11 comorbid_sp___11 HIV positive<br>12 comorbid_sp___12 CP or developmental disability<br>13 comorbid_sp___13 No comorbid conditions
283	[unk_110]	If the above question cannot be answered, please check the box here:	checkbox<br>1 unk_110___1 Unknown
284	[gg_tot_adm_sp]	Total GG score at admission to acute rehab:	text (number)
285	[unk_111]	If the above question cannot be answered, please check the box here:	checkbox<br>1 unk_111___1 Unknown
286	[gg_tot_disch_sp]	Total GG score at discharge from acute rehab:	text (number)
287	[unk_112]	If the above question cannot be answered, please check the box here:	checkbox<br>1 unk_112___1 Unknown
288	[gg_mot_adm_sp]	Mobility GG score at admission to acute rehab:	text (number)
289	[unk_113]	If the above question cannot be answered, please check the box here:	checkbox<br>1 unk_113___1 Unknown
290	[gg_mot_disch_sp]	Mobility GG score at discharge from acute rehab:	text (number)
291	[unk_114]	If the above question cannot be answered, please check the box here:	checkbox<br>1 unk_114___1 Unknown
292	[skin_incidence_sp]	Pressure wounds, number of locations affected during acute rehab admission: If more than 4 pressure wounds, report 4 most severe	dropdown<br>1 0<br>2 1<br>3 2<br>4 3<br>5 4
293	[unk_115]	If the above question cannot be answered, please check the box here:	checkbox<br>1 unk_115___1 Unknown
294	[skin_stage_1_sp]<br>Show the field ONLY if:<br>[skin_incidence] = '2' or [skin_incidence] = '3' or [skin_incidence] = '4' or [skin_incidence] = '5'	Pressure wounds, wound stage of the first location:	dropdown<br>1 1<br>2 2<br>3 3<br>4 4<br>5 Unstageable
295	[unk_116]<br>Show the field ONLY if:<br>[skin_incidence] = '2' or [skin_incidence] = '3' or [skin_incidence] = '4' or [skin_incidence] = '5'	If the above question cannot be answered, please check the box here:	checkbox<br>1 unk_116___1 Unknown

	296	[skin_stage_2_sp]<br><br>Show the field ONLY if:<br>[skin_incidence] = '5' or [skin_incidence] = '4' or [skin_incidence] = '3'	Pressure wounds, wound stage of the second location:	dropdown<br>1 1<br>2 2<br>3 3<br>4 4<br>5 Unstageable
	297	[unk_117]<br><br>Show the field ONLY if:<br>[skin_incidence] = '5' or [skin_incidence] = '4' or [skin_incidence] = '3'	If the above question cannot be answered, please check the box here:	checkbox<br>1 unk_117___1 Unknown
	298	[skin_stage_3_sp]<br><br>Show the field ONLY if:<br>[skin_incidence] = '5' or [skin_incidence] = '4'	Pressure wounds, wound stage of the third location:	dropdown<br>1 1<br>2 2<br>3 3<br>4 4<br>5 Unstageable
	299	[unk_118]<br><br>Show the field ONLY if:<br>[skin_incidence] = '5' or [skin_incidence] = '4'	If the above question cannot be answered, please check the box here:	checkbox<br>1 unk_118___1 Unknown
	300	[skin_stage_4_sp]<br><br>Show the field ONLY if:<br>[skin_incidence] = '5'	Pressure wounds, wound stage of the fourth location:	dropdown<br>1 1<br>2 2<br>3 3<br>4 4<br>5 Unstageable
	301	[unk_119]<br><br>Show the field ONLY if:<br>[skin_incidence] = '5'	If the above question cannot be answered, please check the box here:	checkbox<br>1 unk_119___1 Unknown

	302	[skin_location_1_sp]<br><br>Show the field ONLY if:<br>[skin_incidence] = '2' or [skin_incidence] = '3' or [skin_incidence] = '4' or [skin_incidence] = '5'	Pressure wounds, location of the first wound:	dropdown<br>1 Face<br>2 Occiput<br>3 Ear (Left)<br>4 Ear (Right)<br>5 Shoulder (Left)<br>6 Shoulder (Right)<br>7 Cervical spine<br>8 Elbow (Left)<br>9 Elbow (Right)<br>10 Thoracic spine<br>11 Sternum<br>12 Anterior hips (Left)<br>13 Anterior hips (Right)<br>14 Low back/PSIS (Left)<br>15 Low back/PSIS (Right)<br>16 Ischium (Left)<br>17 Ischium (Right)<br>18 Buttock (Left)<br>19 Buttock (Right)<br>20 Sacrum<br>21 Coccyx<br>22 Knee (Left)<br>23 Knee (Right)<br>24 Leg (Left)<br>25 Leg (Right)<br>26 Heel (Left)<br>27 Heel (Right)<br>28 Medial malleolus (Left)<br>29 Medial malleolus (Right)<br>30 Lateral malleolus (Left)<br>31 Lateral malleolus (Right)<br>32 Foot (Left)<br>33 Foot (Right)<br>34 Toes (Left)<br>35 Toes (Right)
	303	[unk_120]<br><br>Show the field ONLY if:<br>[skin_incidence] = '2' or [skin_incidence] = '3' or [skin_incidence] = '4' or [skin_incidence] = '5'	If the above question cannot be answered, please check the box here:	checkbox<br>1 unk_120___1 Unknown

	304	[skin_location_2_sp]<br><br>Show the field ONLY if:<br>[skin_incidence] = '3' or [skin_incidence] = '4' or [skin_incidence] = '5'	Pressure wounds, location of the second wound:	dropdown<br>1 Face<br>2 Occiput<br>3 Ear (Left)<br>4 Ear (Right)<br>5 Shoulder (Left)<br>6 Shoulder (Right)<br>7 Cervical spine<br>8 Elbow (Left)<br>9 Elbow (Right)<br>10 Thoracic spine<br>11 Sternum<br>12 Anterior hips (Left)<br>13 Anterior hips (Right)<br>14 Low back/PSIS (Left)<br>15 Low back/PSIS (Right)<br>16 Ischium (Left)<br>17 Ischium (Right)<br>18 Buttock (Left)<br>19 Buttock (Right)<br>20 Sacrum<br>21 Coccyx<br>22 Knee (Left)<br>23 Knee (Right)<br>24 Leg (Left)<br>25 Leg (Right)<br>26 Heel (Left)<br>27 Heel (Right)<br>28 Medial malleolus (Left)<br>29 Medial malleolus (Right)<br>30 Lateral malleolus (Left)<br>31 Lateral malleolus (Right)<br>32 Foot (Left)<br>33 Foot (Right)<br>34 Toes (Left)<br>35 Toes (Right)
	305	[unk_121]<br><br>Show the field ONLY if:<br>[skin_incidence] = '3' or [skin_incidence] = '4' or [skin_incidence] = '5'	If the above question cannot be answered, please check the box here:	checkbox<br>1 unk_121___1 Unknown

	306	[skin_location_3_sp]<br><br>Show the field ONLY if:<br>[skin_incidence] = '4' or [skin_incidence] = '5'	Pressure wounds, location of the third wound:	dropdown<br>1 Face<br>2 Occiput<br>3 Ear (Left)<br>4 Ear (Right)<br>5 Shoulder (Left)<br>6 Shoulder (Right)<br>7 Cervical spine<br>8 Elbow (Left)<br>9 Elbow (Right)<br>10 Thoracic spine<br>11 Sternum<br>12 Anterior hips (Left)<br>13 Anterior hips (Right)<br>14 Low back/PSIS (Left)<br>15 Low back/PSIS (Right)<br>16 Ischium (Left)<br>17 Ischium (Right)<br>18 Buttock (Left)<br>19 Buttock (Right)<br>20 Sacrum<br>21 Coccyx<br>22 Knee (Left)<br>23 Knee (Right)<br>24 Leg (Left)<br>25 Leg (Right)<br>26 Heel (Left)<br>27 Heel (Right)<br>28 Medial malleolus (Left)<br>29 Medial malleolus (Right)<br>30 Lateral malleolus (Left)<br>31 Lateral malleolus (Right)<br>32 Foot (Left)<br>33 Foot (Right)<br>34 Toes (Left)<br>35 Toes (Right)
	307	[unk_122]<br><br>Show the field ONLY if:<br>[skin_incidence] = '4' or [skin_incidence] = '5'	If the above question cannot be answered, please check the box here:	checkbox<br>1 unk_122___1 Unknown

	308	[skin_location_4_sp]<br>Show the field ONLY if:<br>[skin_incidence] = '5'	Pressure wounds, location of the fourth wound:	dropdown<br>1 Face<br>2 Occiput<br>3 Ear (Left)<br>4 Ear (Right)<br>5 Shoulder (Left)<br>6 Shoulder (Right)<br>7 Cervical spine<br>8 Elbow (Left)<br>9 Elbow (Right)<br>10 Thoracic spine<br>11 Sternum<br>12 Anterior hips (Left)<br>13 Anterior hips (Right)<br>14 Low back/PSIS (Left)<br>15 Low back/PSIS (Right)<br>16 Ischium (Left)<br>17 Ischium (Right)<br>18 Buttock (Left)<br>19 Buttock (Right)<br>20 Sacrum<br>21 Coccyx<br>22 Knee (Left)<br>23 Knee (Right)<br>24 Leg (Left)<br>25 Leg (Right)<br>26 Heel (Left)<br>27 Heel (Right)<br>28 Medial malleolus (Left)<br>29 Medial malleolus (Right)<br>30 Lateral malleolus (Left)<br>31 Lateral malleolus (Right)<br>32 Foot (Left)<br>33 Foot (Right)<br>34 Toes (Left)<br>35 Toes (Right)
	309	[unk_123]<br>Show the field ONLY if:<br>[skin_incidence] = '5'	If the above question cannot be answered, please check the box here:	checkbox<br>1 unk_123___1 Unknown
	310	[disp_sp]	Please indicate discharge disposition from the acute rehab unit:	dropdown<br>1 Home<br>2 Acute Hospital<br>3 SAR<br>4 LTAC<br>5 Hospice<br>6 Deceased
	311	[unk_124]	If the above question cannot be answered, please check the box here:	checkbox<br>1 unk_124___1 Unknown

	312	[skin_project_complete]	Section Header: *Form Status*<br>Complete?	dropdown<br>0 Incomplete<br>1 Unverified<br>2 Complete
**Instrument: Rehab LOS and Multiple Admissions (draft_multiple_rehab_admissions)**				
	313	[rehab_date_1]	Indicate the patient's initial date of admission to acute rehab:	text (date_mdy), Required, Identifier
	314	[transf_number]	Please indicate the number of times this patient transferred from and later was readmitted to acute rehab:	radio, Required<br>1 1<br>2 2<br>3 3<br>4 4 or more<br>5 N/A
	315	[tot_los_rehab]	Total length of stay in acute rehab (sum of days across all admissions):	text (number), Required
	316	[los_acutecare]<br>Show the field ONLY if:<br>[transf_number] = '1'	Length of stay in acute care for the first transfer from acute rehab:	text
	317	[los_acutecare_2]<br>Show the field ONLY if:<br>[transf_number] = '2'	Length of stay in acute care for the second transfer from acute rehab:	text (number)
	318	[los_acutecare_3]<br>Show the field ONLY if:<br>[transf_number] = '3'	Length of stay in acute care for the third transfer from acute rehab:	text (number)
	319	[los_acutecare_4]<br>Show the field ONLY if:<br>[transf_number] = '4'	Length of stay in acute care for the fourth transfer from acute rehab:	text (number)
	320	[transf_reason]<br>Show the field ONLY if:<br>[transf_number] = '1'	Please indicate the reason for transfer to acute care for the first transfer from acute rehab:	checkbox<br>1 transf_reason___1 Respiratory Distress<br>2 transf_reason___2 Sepsis<br>3 transf_reason___3 Cardiac Distress<br>4 transf_reason___4 AMS<br>5 transf_reason___5 Bleeding
	321	[transf_reason_2]<br>Show the field ONLY if:<br>[transf_number] = '2'	Please indicate the reason for transfer to acute care for the second transfer from acute rehab:	checkbox<br>1 transf_reason_2___1 Respiratory Distress<br>2 transf_reason_2___2 Sepsis<br>3 transf_reason_2___3 Cardiac Distress<br>4 transf_reason_2___4 AMS<br>5 transf_reason_2___5 Bleeding
	322	[transf_reason_3]<br>Show the field ONLY if:<br>[transf_number] = '3'	Please indicate the reason for transfer to acute care for the third transfer from acute rehab:	checkbox<br>1 transf_reason_3___1 Respiratory Distress<br>2 transf_reason_3___2 Sepsis<br>3 transf_reason_3___3 Cardiac Distress<br>4 transf_reason_3___4 AMS<br>5 transf_reason_3___5 Bleeding
	323	[transf_reason_4]<br>Show the field ONLY if:<br>[transf_number] = '4'	Please indicate the reason for transfer to acute care for the fourth transfer from acute rehab:	checkbox<br>1 transf_reason_4___1 Respiratory Distress<br>2 transf_reason_4___2 Sepsis<br>3 transf_reason_4___3 Cardiac Distress<br>4 transf_reason_4___4 AMS<br>5 transf_reason_4___5 Bleeding

	324	[bim_adm2]<br><br>Show the field ONLY if:<br>[transf_number] = '1'	BIM score at the second admission to acute rehab:	text (number)
	325	[bim_adm3]<br><br>Show the field ONLY if:<br>[transf_number] = '2'	BIM score at the third admission to acute rehab:	text (number)
	326	[bim_adm4]<br><br>Show the field ONLY if:<br>[transf_number] = '3'	BIM score at the fourth admission to acute rehab:	text (number)
	327	[bim_adm5]<br><br>Show the field ONLY if:<br>[transf_number] = '4'	BIM score at the fifth admission to acute rehab:	text (number)
	328	[gg_total_adm2]<br><br>Show the field ONLY if:<br>[transf_number] = '1'	Total GG score at the second admission to acute rehab:	text (number)
	329	[gg_total_adm3]<br><br>Show the field ONLY if:<br>[transf_number] = '2'	Total GG score at the third admission to acute rehab:	text (number)
	330	[gg_total_adm4]<br><br>Show the field ONLY if:<br>[transf_number] = '3'	Total GG score at the fourth admission to acute rehab:	text (number)
	331	[gg_total_adm5]<br><br>Show the field ONLY if:<br>[transf_number] = '4'	Total GG score at the fifth admission to acute rehab:	text (number)
	332	[gg_motor_adm2]<br><br>Show the field ONLY if:<br>[transf_number] = '1'	Mobility GG score at the second admission to acute rehab:	text (number)
	333	[gg_motor_adm3]<br><br>Show the field ONLY if:<br>[transf_number] = '2'	Mobility GG score at the third admission to acute rehab:	text (number)
	334	[gg_motor_adm4]<br><br>Show the field ONLY if:<br>[transf_number] = '3'	Mobility GG score at the fourth admission to acute rehab:	text (number)
	335	[gg_motor_adm5]<br><br>Show the field ONLY if:<br>[transf_number] = '4'	Mobility GG score at the fifth admission to acute rehab:	text (number)
	336	[note_disch]	NOTE: No discharge scores from multiple transfers/discharges will be collected. Only the discharge scores from the final discharge from acute rehab, already collected on the Retrospective Data form, will be used.	descriptive
	337	[draft_multiple_rehab_admissions_complete]	Section Header: *Form Status*<br>Complete?	dropdown<br>0 Incomplete<br>1 Unverified<br>2 Complete
